# Determination of the optimal inspiratory pressure providing adequate ventilation while minimizing gastric insufflation using real-time ultrasonography in Chinese children: a prospective, randomized, double-blind study

**DOI:** 10.1186/s12871-017-0417-0

**Published:** 2017-09-11

**Authors:** Xiaowei Qian, Qiong Hu, Hang Zhao, Bo Meng, Yang Nan, Hong Cao, Qingquan Lian, Jun Li

**Affiliations:** 1 Department of Anesthesiology, Women’s Hospital, School of Medicine, Zhejiang University, Hangzhou, China; 2 Department of Anesthesiology, Critical Care and Pain Medicine, The Second Affiliated Hospital and Yuying Children Hospital of Wenzhou Medical University, West College Road 109, Wenzhou, 325027 China; 3 Department of Anesthesiology, Ningbo Women and Children’s Hospital, Ningbo, China; 4 Department of Anesthesiology, The Second People’s Hospital of Ningbo, Ningbo, China

**Keywords:** Gastric insufflation, Antral area, Ultrasonography

## Abstract

**Background:**

During facemask ventilation, gastric insufflation is defined as appearance of a comet-tail or an acoustic shadow on ultrasonography. Ultrasonographic measurement of antral cross-section area (CSA) may reflect an insufflated antrum and provide interesting semi-quantitative data in regard to the gastric insufflation. This study aimed to determine the appropriate level of inspiratory pressure sufficient to provide adequate pulmonary ventilation with a lower occurrence of gastric insufflation during facemask pressure-controlled ventilation using real-time ultrasonography in paralyzed children.

**Methods:**

Ninety children, ASA I-II, aged from 2 to 4 years, scheduled for general anesthesia were enrolled in this randomized and double-blinded study. Children were randomized into one of the five groups (P8, P10, P12, P14, and P16) defined by the applied inspiratory pressure during facemask ventilation: 8, 10, 12, 14, and 16 cm H_2_O. Anesthesia induction was conducted with fentanyl and propofol. Rocuronium was administrated as a muscle relaxant. After rocuronium administration, facemask ventilation was performed for 120 s. Gastric insufflation (GI+) was detected by ultrasonography, and the antral CSA before and after facemask ventilation were also measured using ultrasonography. Respiratory variables were monitored.

**Results:**

Gastric insufflation was detected in 32 children (3/18 in group P8, 5/18 in group P10, 7/18 in group P12, 8/16 in group P14, and 9/14 in group P16). The antral CSA after facemask ventilation statistically increased in subgroups P14 GI+ and P16 GI+ for whom gastric insufflation was detected by ultrasonography, whereas it did not change statistically in other groups. Lung ventilation was inadequate for group P8 or P10.

**Conclusion:**

We concluded that an inspiratory pressure of 12 cm H_2_O is sufficient to provide adequate ventilation with a lower occurrence of gastric insufflation during induction of general anesthesia in paralyzed Chinese children aged from 2 to 4 years old.

**Trial registration:**

(ChiCTR-IPR-16007960). Registered 21 February 2016

**Conclusion heading:** Ultrasound for determining gastric insufflation

## Background

Aspiration of gastric contents is one of the most feared perioperative complication, and may result in significant morbidity and mortality [1, 2]. During the induction of general anesthesia, one critical risk factor for aspiration is gastric insufflation during facemask ventilation (FMV) [3]. Although pulmonary aspiration is profoundly decreased in modern anesthesia in children [4], the time of FMV for anesthesia induction is still the most vulnerable period for gastric regurgitation following a gastric insufflation. Thus, we have to try to apply a safer ventilation mode and appropriate inspiratory pressure to minimize the risk of gastric insufflation consistently.

In adult patients, the risk of gastric insufflation and consequently the adverse complications are significantly reduced with pressure-controlled FMV compared with manual or volume-controlled FMV [5]. Using the traditional auscultation method, previous study determined the inspiratory pressure for preventing gastric insufflation during FMV to be 20 cm H_2_O in adult patients [6]. In children, an inspiratory pressure less than 15 cm H_2_O determined by the auscultation method can provide safe ventilation without gastric insufflation during pressure-controlled FMV [7].

Conventionally, stethoscopic auscultation has been used to evaluate the gastric insufflation during ventilation. Recently, two studies reported that ultrasonography could detect air in stomach more precisely compared with stethoscopic auscultation during facemask ventilation [8, 9]. Using real-time ultrasonography, air entry into stomach can be detected with acoustic shadows or comet-tail appearance that appears in the gastric antrum, which provides a qualitative examination of gastric insufflation [8, 9]. Recent studies have shown that ultrasonographic measurement of antral cross-section area (CSA) was a quantitative approach to determine gastric insufflation during facemask ventilation [8–11]. Bouvet et al. reported that a threshold inspiratory pressure for reducing gastric insufflation with proper ventilation during FMV to be 15 cm H2O in nonparalyzed adults in their study by ultrasonographic measurement of antral CSA [8].

In this prospective study, we evaluated the appropriate level of inspiratory pressure that allows adequate lung ventilation with a lower occurrence of gastric insufflation during FMV using real-time ultrasonography in paralyzed children.

## Methods

Ethical approval for this study was provided by the Ethical Committee (ref: C2016-07) of the Second Affiliated Hospital and Yuying Children Hospital of Wenzhou Medical University (Wenzhou, China) on 15 February 2016. The present study was registered at Chinese Clinical Trials.gov (ChiCTR-IPR-16007960) and was performed in the Second Affiliated Hospital and Yuying Children Hospital of Wenzhou Medical University, from 01 March 2016 to 01 July 2016. After obtaining written informed consent from all parents, 90 children, ASA physical status I or II, aged from 2 to 4 years, scheduled for high ligation of hernial sac or sheath under general anesthesia were enrolled in this prospective, randomized, double-blinded study. Exclusion criteria were parental refusal, a known or predicted respiratory disease, a predicted difficult intubation, oropharyngeal or facial pathology, full stomach or emergency surgery, and body mass index above 35 kg/m^2^.

### Study design

Using a computer-generated list, children were randomized into one of the five groups (P8, P10, P12, P14, and P16) defined by the applied inspiratory pressure during FMV: 8, 10, 12, 14, and 16 cm H_2_O. Blocking, stratification, or other restrictions were not used when group assignments were created. Children were allocated using a 1:1 ratio. Sealed and coded opaque envelopes were used to ensure allocation concealment. All these above works were performed by an anesthesiologist who did not participate in the procedure of anesthesia. The anesthesiologist performing ultrasound assessment was blinded to group allocation and was the same as the one assessing the antral CSA during facemask ventilation.

**Fig. 1:**
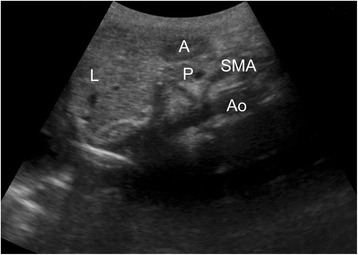
Sagittal sonographic image of the gastric antrum. A = antrum; P = pancreas; L = liver; SMA = superior mesenteric artery; Ao = aorta

**Fig. 2:**
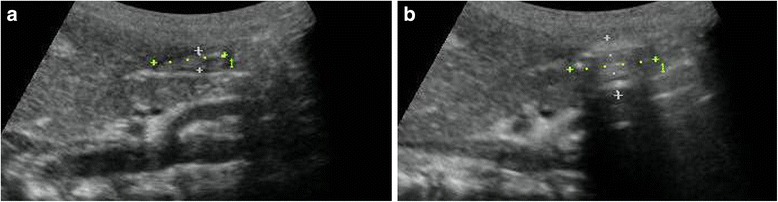
Antral images (a) Before facemask ventilation. (b) After gastric insufflation with an internal “acoustic shadow or comet-tail appearance”

All children were premedicated with 0.5 mg/kg midazolam orally 30 min before induction of anesthesia. We allowed parents to accompany their children until an intravenous catheter was inserted under local anesthesia using EMLA (Eutectic Mixture of Local Anesthetics) cream (AstraZeneca, UK). A skilled nurse performed all the punctures avoiding repeated puncture. In the operation room, continuous pulse oximetry, electrocardiogram, and non-invasive arterial pressure were measured. In this study, we defined the significant increase in antral CSA after FMV as gastric insufflation using an ultrasonography (SonoSite, Inc., Bothwell, WA) with a 2- to 5-MHz convex transducer. All children were lying in the supine position on the operation table. In brief, using the aorta and the superior mesenteric vein as internal landmarks, longitudinal (D1) and anteroposterior (D2) diameters of the gastric antrum in cross-section on a sagittal plane were measured (Figs. 1 and 2) as described previously [8, 12]. Measurements were repeated three times. The antral CSA was calculated using the following formula previously used by Bouvet et al. [8]: Antral area = π × D1 × D2/4.

After 2 min of preoxygenation using a ventilator circuit (Primus; Dräger, Lübeck, Germany), general anesthesia was conducted with fentanyl 2 μg/kg and propofol 3 mg/kg administrated over 45 s. Spontaneous breathing was maintained while the fentanyl and propofol was given. Immediately after fentanyl and propofol administration, rocuronium 0.6 mg/kg was given and an oropharyngeal airway was inserted and a well fitting facemask was fixed with two hands by an experienced anesthesiologist who was blinded to the results of perioperative ultrasonographic measurement of antral CSA. FMV was delivered for 120 s with a frequency of 20 breaths per min, and an inspiratory-to-expiratory ratio of 1:2, a flow of 100% oxygen 3 L/min. An applied inspiratory pressure was set according to the corresponding group. No positive end-expiratory pressure was applied. The trachea was intubated after the mask ventilation. After the trachea was intubated, real-time ultrasonographic measurement of antral CSA was performed again. In this current study, we defined the significant increase in antral CSA after FMV as gastric insufflation. GI+ was defined as gastric insufflation detected by ultrasonography, whereas GI- was defined as gastric insufflation not detected by ultrasonography.

The following respiratory variables were recorded using an Anaesthesia monitor at time 30, 60, 90, and 120 s during FMV: pulse oximetry (SpO_2_, %), the tidal volume (Vt, ml/kg), and the end-tidal carbon dioxide concentration in the expired air (EtCO_2_, mmHg). Adequate ventilation was defined as Vt greater than 7 ml/kg according to Lagarde et al. [7].

### Statistical and power analysis

In our preliminary study, we found that gastric insufflation occurred in 17% (1 of 6 patients), 33% (2 of 6 patients), 40% (2 of 5 patients), 67% (4 of 6 patients), and 80% (4 of 5 patients) of patients for the applied inspiratory pressure 8, 10, 12, 14, and 16 cm H_2_O during FMV. The Cochran-Armitage test for the trend in proportions was used to calculate the sample size. We assumed that the applied inspiratory pressure 8, 10, 12, 14, and 16 cm H_2_O would produce proportions of gastric insufflation of 15, 30, 45, 60, and 75%, respectively. The total sample of 65 subjects (13 per group) are required to achieve 95% power to detect a linear trend using a two-sided Z test with continuity correction and a significance level of 0.05 (PASS^*^ 11.0; NCSS, LCC, Kaysville, UT). We assigned 90 subjects to allow for the possibility of rejected cases.

Data were analyzed by the Statistical Package for Social Science program, version 16.0 (SPSS^*^, Chicago, IL). Demographics (age, weight, height, and body mass index) were presented as mean ± SD and analyzed by one-way ANOVA. The chi-square trend test (linear-by-linear association) was used to analyze the occurence of gastric insufflation in the five groups. The antral CSA before and after FMV were expressed as median (interquartile range) and were compared by means of Wilcoxon matched-pair tests. The Bonferroni’s correction was used to get adjusted *p* values.

Using the Statistica® software package (Release 6.0, StatSoft Inc., Tulsa, OK, USA), we analyzed the respiratory variables (Vt and EtCO_2_) by two-way ANOVA, followed by a Bonferroni post hoc test.

## Results

**Fig. 3:**
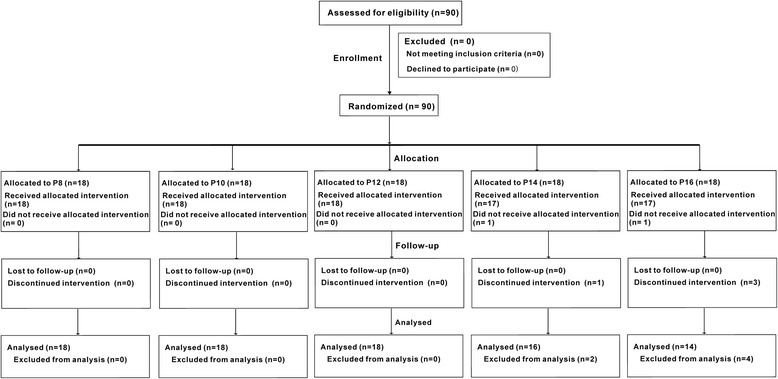
Consort study flow chart

Demographic data are described in Table 1. A total of 90 patients were included. Two patients from group P14 and group P16 were excluded from the study because the antral CSA could not be measured before FMV (Fig. 3). No difference of demographic characteristics was found among groups.

**Table 1:** Patient’s characteristics

	P8 (*n* = 18)	P10 (*n* = 18)	P12 (*n* = 18)	P14 (*n* = 17)	P16 (*n* = 17)
Age (yr)	2.8 ± 0.8	2.8 ± 0.7	2.9 ± 0.8	2.9 ± 0.7	3.1 ± 0.8
Sex ratio (F/M) (n)	7/11	6/12	6/12	7/10	8/9
Weight (kg)	12.9 ± 2.8	13.0 ± 2.3	13.4 ± 1.9	13.5 ± 2.2	13.9 ± 2.2
Height (cm)	92.3 ± 8.9	92.6 ± 7.6	92.8 ± 6.5	93.5 ± 7.8	96.0 ± 7.9
BMI (kg/m^2^)	15.0 ± 1.6	15.1 ± 1.5	15.5 ± 1.3	15.4 ± 1.2	15.1 ± 1.8
CSA (mm^2^)	133.3 ± 48.7	118.8 ± 41.4	135.4 ± 31.8	133.1 ± 43.5	146.6 ± 57.1

Data are expressed as mean ± SD or number. *BMI* body mass index, *CSA* antral cross-section area

**Fig. 4:**
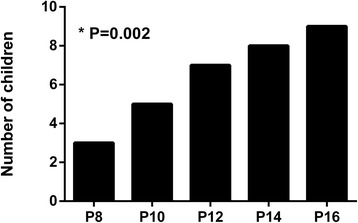
Gastric insufflation detected by ultrasonography in the five groups. *P value is given for chi-square trend test (linear-by-linear association)

Gastric insufflation was detected in 32 children using ultrasonography (3/18 in group P8, 5/18 in group P10, 7/18 in group P12, 8/16 in group P14, and 9/14 in group P16) (Fig. 4).

Three patients from group P16 and one patient from Group P14 were excluded from the study, because the antral CSA could not be measured due to a large air insufflation in the stomach after FMV. As shown in Table 2, there were statistically increases in the antral CSA in subgroups P14 GI+ and P16 GI+. However, there were no statistically increases in subgroups P8 GI+, P8 GI-, P10 GI+, P10 GI-, P12 GI+, P12 GI-, P14 GI-, and P16 GI-.

**Table 2:** Antral cross-sectional area before and after facemask ventilation

	Antral area before facemask ventilation (mm^2^)	Antral area after facemask ventilation (mm^2^)	Adjusted *P* values
P8 Total (*n* = 18)	123.7 (97.7-173.5)	138.2 (100.1-190.0)	0.999
P8 GI+ (*n* = 3)	173.5 (98.9-173.5)	208.9 (150.7-247.4)	0.327
P8 GI- (*n* = 15)	117.8 (94.2-150.7)	129.5 (94.2-150.7)	0.498
P10 Total (*n* = 18)	113.8 (87.1-134.8)	133.9 (88.3 -194.9)	0.588
P10 GI+ (*n* = 5)	120.9 (104.4-146.8)	193.9 (162.1-236.4)	0.129
P10 GI- (*n* = 13)	98.9 (84.8-140.1)	94.2 (76.6-144.5)	0.621
P12 Total (*n* = 18)	123.3 (111.7-164.9)	162.2 (116.4-197.7)	0.051
P12 GI+ (*n* = 7)	131.9 (120.9-186.9)	208.9 (183.8-289.0)	0.054
P12 GI- (*n* = 11)	117.8 (102.1-169.6)	125.6 (102.1-160.2)	0.715
P14 Total (*n* = 16)	121.3 (96.2-158.4)	164.9 (108.0-217.4)	0.102
P14 GI+ (*n* = 8)	131.9 (104.6-181.4)	210.8 (175.5-285.6)	0.036
P14 GI- (*n* = 8)	120.9 (84.8-152.1)	112.3 (86.5-150.7)	0.618
P16 Total (*n* = 14)	126.0 (77.7-189.6)	206.9 (127.2-278.0)	0.021
P16 GI+ (*n* = 9)	129.6 (111.5-197.9)	276.5 (207.0-294.9)	0.024
P16 GI- (*n* = 5)	77.8 (76.6-170.0)	84.8 (81.3-157.5)	0.465

Data are expressed as median (interquartile range). The Bonferroni’s correction was used for the adjusted *P* values. *GI−* gastric insufflation not detected by ultrasonography, *GI+* gastric insufflation detected by ultrasonography

**Fig. 5:**
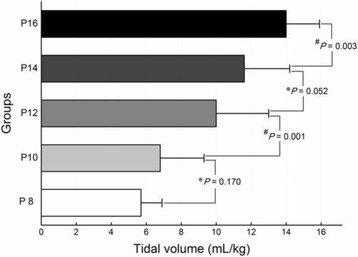
Mean tidal volume during facemask ventilation. Vertical bars represent 95% Bonferroni-corrected CIs

The mean value of Vt at time 30, 60, 90, and 120 s among the groups were compared. There was a statistically difference among the five groups (Fig. 5). Vt increased in group P12 (10.0 ± 3.0 ml/kg) in comparison with group P10 (6.8 ± 2.5 ml/kg) (*P* = 0.000). An inspiratory pressure of 8 or 10 cm H_2_O was not sufficient to provide a Vt >7 ml/kg. No difference was found between groups P8 and P10, and between groups P12 and P14. SpO_2_ was greater than 98% in all children during FMV.

**Fig. 6:**
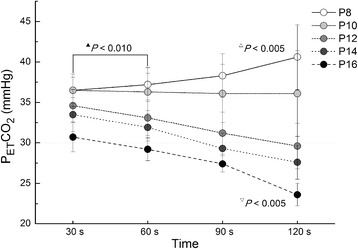
End-tidal carbon dioxide concentration during facemask ventilation. Vertical bars represent 95% Bonferroni-corrected CIs. ^▲^ *P* < 0.01 between group P8 and all other groups at time 30 and 60s, between group P10 and all other groups at time 30 and 60s; ^∆^ *P* < 0.005 between group P8 and all other groups at time 120 s; ^∇^ *P* < 0.005 between group P16 and all other groups at time 120 s

EtCO_2_ significantly increased in groups P8 and P10 compared to that of other groups at time 30 and 60s (*P* < 0.01). In group P8, EtCO_2_ was 40.6 ± 4.0 mmHg that was significantly higher than that in all other groups at time 120 s (*P* < 0.005), whereas EtCO_2_ significantly decreased in group P16 (23.6 ± 1.4 mmHg) as compared to other groups (*P* < 0.005) (Fig. 6).

## Discussion

By using ultrasonography, the occurrence of gastric insufflation statistically increased with inspiratory pressure after facemask ventilation, with a threshold of 12 cm H_2_O above which the occurrence of gastric insufflation became statistically significant. In this study, we found that an inspiratory pressure of 12 cm H_2_O was sufficient to provide adequate ventilation with a lower occurrence of gastric insufflation during induction of general anesthesia in paralyzed Chinese children aged from 2 to 4 years old.

One previous study showed that gastric insufflation occurs during FMV in children, and the inspiratory pressure threshold for gastric insufflation depends on age [7]. It was recommended not to use inspiratory pressure larger than 15 cm H_2_O to prevent gastric insufflation in children. However, the gastric insufflation was evaluated by epigastric auscultation which did not provide a precise detection of gastric inflation volume [7]. Recent studies suggested that ultrasonographic measurement of the antral area can be used to assess the gastric content and volume [8–11, 13, 14]. Stethoscopic auscultation is less sensitive method compared with ultrasonography in detecting gastric insufflation during FMV [8]. Park et al. also indicated that ultrasonography was more sensitive in determining gastric insufflation than the epigastric auscultation [9].

In the present study, gastric insufflation occurred in 64% of patients when the inspiratory pressure 16 cm H_2_O was applied, which is consistent with the study conducted by Lagarde et al., indicating that gastric insufflation occurred in >58% of cases when the inspiratory pressure > 15 cm H_2_O [7]. This is different from the study for adult patient, where only 35% of adult patients during FMV with an inspiratory pressure 15 cm H_2_O have gastric insufflation [8]. We think the difference could be partially attributed to the usage of nondepolarizing muscle relaxants in the present study, because nondepolarizing muscle relaxants could decrease the resting tone in the upper esophageal sphincter muscle and impair the function of pharyngeal muscle in children [15, 16]. In addition, compared with adults, the esophageal sphincter muscle is not mature in children and the esophageal sphincter tone is lower than adults [7, 9, 17].

Our results showed that the occurence of gastric insufflation was lower in group P8 and P10. However, inspiratory pressure of 8 or 10 cm H_2_O for the FMV is rarely used in clinical practice, because it will result in inadequate ventilation. Indeed, an inspiratory pressure of 8 or 10 cm H_2_O was not sufficient to provide a Vt >7 ml/kg and the EtCO2 was significantly higher in group P8 and P10. However, SpO_2_ was greater than 98% during FMV in all children. An inspiratory pressure of 12 cm H_2_O provided less occurence of gastric insufflation than that for a pressure of 14 and 16 cm H_2_O. An inspiratory pressure of 12 cm H_2_O is sufficient to provide adequate ventilation.

Ultrasonographic measurement of antral cross-section area (CSA) may provide interesting quantitative data in regard to the gastric insufflation during FMV. Our data also showed that the antral CSA after facemask ventilation statistically increased only in subgroups P14GI+ and P16GI+ for whom gastric insufflation was detected by ultrasonography. However, it had been suggested that increased CSA on ultrasonography may not directly mean that gastric insufflation occur [9]. In small children, the difficulties in fixing the probe in proper site, the pressure from the probe placement, and the changes in the gastric position following neuromuscular blockade may result in a bias of ultrasonography images [9]. Large sample investigations may be needed to solve this problem.

Although manual ventilation is commonly used during facemask ventilation for anesthesia induction in clinical practice, pressure-controlled FMV provides lower inspiratory pressure compared with manual ventilation at a similar tidal volume which results in reduced occurrence of gastric insufflation [5, 7, 18]. von Goedecke et al. indicated that pressure-controlled FMV had a lower inspiratory peak flow rates and peak airway pressures compared with manual ventilation, which provided a safer facemask ventilation mode [5]. Seet et al. also reported that pressure-controlled FMV resulted in a lower peak airway pressures which may improve the patient safety [18]. Based on these previous studies, pressure-controlled FMV mode was selected in this present study. However, more recently, Park et al. compared the occurrence of gastric insufflation in paralysed children during pressure-controlled FMV and manual ventilation [9]. Although the peak airway pressures in pressure-controlled FMV group was lower than that in manual ventilation group, the occurrence of gastric insufflation was only slightly lower during pressure-controlled FMV than during manual ventilation which has no statistically difference. The authors suggested that the small sample size may result in this meaningless and the occurrence of gastric insufflation may not be absolutely prevented even in pressure-controlled FMV with low peak airway pressures in paralyzed children.

Adverse events such as aspiration and desaturation events were not found in the present study. Although antral CSA after facemask ventilation statistically increased in subgroups P14GI+ and P16GI+, aspiration events were not occurred in both groups. However, increased inspiratory pressure results in a higher occurrence of gastric insufflation which may increase the risk of aspiration. The lung ventilation was inadequate in group P8 and P10, but it did not result in desaturation events. This may be explained by the administration of 100% oxygen and by minute ventilation almost reaching normal values. In addition, insufficient sample size may be a cause of no statistical difference in adverse events.

This present study had some limitations. Firstly, increased antral CSA on ultrasonography might not necessarily mean gas insufflation and increased risk of aspiration, because the pressure from the probe placement and the use of neuromuscular blockade might result in a bias of ultrasonography images. Secondly, ultrasonography could not be used to quantify antral CSA due to marked gastric insufflation with subsequent patient exclusion from the analysis. Thirdly, the respiratory parameters to determine the ventilator adequacy in this study should be more detailed. We did not monitor the respiratory parameters after intubation and further study may be needed. Lastly, we did not record adverse event such as cardiovascular hemodynamic instability during FMV and further investigation may be needed.

## Conclusions

In summary, using real-time ultrasonography, we demonstrated that an inspiratory pressure of 12 cm H_2_O is sufficient to provide adequate ventilation with a lower occurence of gastric insufflation during pressure-controlled FMV in 2- to 4- year-old paralyzed Chinese children. Increasing inspiratory pressure above this value will result in increased occurence of gastric insufflation.

## Abbreviations

BMI: Body mass index
CSA: Cross-section area
EMLA: Eutectic Mixture of Local Anesthetics
EtCO_2_: The end-tidal carbon dioxide concentration in the expired air
FMV: Facemask ventilation
GI: Gastric insufflation
SpO_2_: Pulse oximetry
Vt: The tidal volume
